# The Conditions of Health: Can We Secure Them ?

**Published:** 1924-04

**Authors:** 


### THE CONDITIONS OF HEALTH.

### CAN WE SECURE THEM ?

On February 19 Dr. Ethel Bentham delivered a lecture, at Sheffield, in connection with the winter's course on "Health Education" upon "The Essentials of Health."

### What is Health ?

I should define health, she said, as such a state of harmony of mind and body that all our powers and functions can be used to the full with enjoyment, and without other limit than the hours of the day. And the simple, practical test that I should suggest is that of being ready and happy to get up in the morning. If you accept that as a test—and it is surely a fair one—one has to realise that but few of us would pass it, that the majority of the people we meet with in our comings and goings are evidently and deplorably below that standard.

### The Proper Function of the Doctor.

Rarely does one find any appreciation of the fact that the proper function of the doctor is to prevent illness; that there are elementary rules of health which are comprehensible and can be studied, and that the doctor can, with the intelligent co-operation of the patient, help to elucidate individual variations and departures from rule. I shall be told that it is hard to expect the general public to put health precepts into practice when their natural guides, the doctors, are still disputing among themselves about the orgin and treatment of so many diseases, and cannot tell for certain what causes cancer or how to cure it—can neither cure a common cold nor prevent influenza or measles ravaging the country every little while. The doctors can answer, too, that there is a large body of knowledge which accumulated in the past, but which is now general knowledge; that the application of much of this is definitely outside the power of the doctor; that they can, at best, only tell you what is well to be done and leave you as individuals and citizens to do it.

### The Conditions of Health.

The actual needs of the body—the conditions of health—are known to everybody and can be stated in a sentence. There must be sufficient air, light and warmth, sufficient appropriate food; there must be opportunity for the organism to exercise its functions freely (that is, to work), and some interest or stimulus for it to do so; and there must, if the health of the mind is to be maintained, be a reasonable certainty of the satisfaction of these elementary needs. We know that if every child were suitably lodged and suitably fed it would grow better, be less exposed to infection, and postpone even the inevitable measles to a later and less dangerous age; that it would probably be more resistant to tuberculosis and certainly less frequently exposed to it. We know that if everyone were suitably warmed and clothed, accidents, eye-diseases, curvature of the spine, corns, flat-foot and inflamed toe-joints would be rarer.

### Our Defects in Feeding.

How far do we get fair play ? The majority of infants are not breast-fed, or only for a short time—weeks perhaps. They are fed on every variety of imitation, the very multiplicity of which shows their unsatisfactoriness. There is an inclination for concentrated, sophisticated, one might almost say tortured kinds of food, till the line between drugs and food becomes difficult to draw. Later on in life we still think that the more nourishing food we swallow, the better is our nutrition. We eat too easily, and we eat too often and too hurriedly; and that is not giving free and fair play to our bodies.

### Effects of Fashion.

Take next our ways of securing warmth. For young infants things are improving. Their clothes are neither so heavy nor so hampering as they were, though doubtless they could still be bettered. But for older children and for women we can hardly say as much. One year clothes begin so late and end so early that girls—and boys too—are very ill-protected from changes of temperature; another year, no woman can step on a 'bus without great ingenuity, and her hat brim scores her neighbour's nose when she does get inside. Women's clothes rarely give them both protection and freedom; while as to the children, you may see a little lad (clad during the week with stockings and overcoat) out on Saturdays as a boy-scout, with a flannel shirt and an apology for knickers showing his blue legs to halfway up to his thighs. The most mischievous effects of fashion are seen in foot-gear. No one can compute the disability and suffering caused by bad boots, for nearly everyone suffers in some degree.

### Air and Light.

I have sketched some of the ways in which we habitually offend against the laws of health, even though we have knowledge certain enough to guide us. These are all matters which are largely, though not altogether, within the control of the individual citizen, and almost entirely at the choice of well-to-do people who can have their clothes made for them, can choose their foodstuffs, and pay for advice as to medicaments. But when we come to the consideration of the two most important of the conditions of health—air and light—these for the majority of us are not within our own control. The facts are substantially the same everywhere, with local variations. This town I do not know. I have some experience of many big towns in England, but have made an extensive study of Kensington, where I live; and as it is one of the richest of the London boroughs the picture will not be overdrawn. Kensington is divided into two parts—the South mainly big houses with pockets of poor folk; the North mainly working people with a largish corner where the big houses prevail. A small part of one ward is notorious—always has been; street names have been changed by the authorities and various agencies have tried to reform it, but there is yet much to do. It is the home of casual people of all sorts. Here one would expect a big death rate—and one gets it. But it is not the slum results that are most important. We want rather to consider the condition of the bulk of the people. Two wards are mainly inhabited by those everyday respectable folk who carry on the work of the world—railwaymen, bricklayers, painters, carpenters, 'bus drivers and conductors, etc., the shopkeepers who supply them, and a few doctors and ministers of religion. In one of these there are 26,000 people in 2,817 houses, containing 15,855 rooms—that is, an average of over 9 persons to a house, and of 1.64 to a room. But the doctors and ministers and shopkeepers occupy more than the average, and many rooms have much more than that. In fact, by far the larger number of families live in two rooms only; some big ones in three. These houses were all built for single occupation, and have deep underground kitchens, which are counted as rooms. In the yard-wide area stands the sanitary dustbin common to all the tenants.

### "Demoralising and Degrading."

I am justified in saying that sufficient air and light, and ordinary decent privacy are impossible to a large section of the population; and the means of cleanliness, according to the standards of better-off people, also, and that we all know it but prefer not to think about it. We may not be certain whether want of light, or want of fats, or of accessory food factors is the cause of rickets; or of how tuberculosis originates, or whether pneumonia is due to an organism or to catching colds; but we do know, every one of us, that such conditions as I have described are demoralising and degrading to both body and mind.

### No Royal Road to Health.

I believe that while there is an infinity of work to be done in medical research into the causation, treatment and prevention of definite diseases, yet that the low standard of well-being and efficiency is due to our disregard of well-known laws of health. There is no short cut or royal road to health, but that it depends on giving our bodies—and our minds—a fair chance, and allowing free natural development—sunlight, clean water and air, work, leisure and security—can be stated in a breath; yet these are things out of reach of most of the people. This would imply that the road to health must be engineered by the politician and the teacher rather than the doctor.

